# ERCP PERFORMANCE IN A TERTIARY BRAZILIAN CENTER: FOCUS ON NEW RISK FACTORS, COMPLICATIONS AND QUALITY INDICATORS

**DOI:** 10.1590/0102-672020180001e1348

**Published:** 2018-06-21

**Authors:** Alana Costa BORGES, Paulo César de ALMEIDA, Stella Maria Torres FURLANI, Marcelo de Sousa CURY, Douglas K. PLESKOW

**Affiliations:** 1 Zilda Arns Hospital and Maternity, Gastrointestinal Endoscopy, Fortaleza, CE, Brasil;; 2 State University of Ceará, Center for Health Sciences, Fortaleza, CE, Brasil;; 3 César Cals General Hospital, Gastrointestinal Endoscopy, Fortaleza, CE, Brasil;; 4 SCOPE Gastrointestinal Endoscopy Unit, Gastrointestinal Endoscopy, Campo Grande, MS, Brasil;; 5 Beth Israel Deaconess Medical Center, Center for Advanced Endoscopy, Boston, Massachusetts,USA

**Keywords:** Risk factors, Postoperative complications, Qualityindicators, healthcare, Endoscopy, gastrointestinal., Fatores de risco, Complicações pós-operatórias, Indicadores de qualidade em assistência à saúde, Endoscopia do sistema digestório.

## Abstract

**Background:**

ERCP can lead to complications, which can be prevented by the recognition of risk factors.

**Aim:**

To identify these risk factors, with quality evaluation.

**Methods:**

Retrospective study in a Brazilian hospital in 194 patients, excluding surgically altered anatomy.

**Results:**

211 ERCPs were performed: 97.6% were therapeutic, 83.4% were started by trainees, with deep cannulation rate of 89.6%. Precut was needed in 16.6% of the ERCPs and classic sphincterotomy in 67.3%, with 75.4% of ductal clearance at single session and 8.0% of technical failure. Inacessible papillas ocurred in 2.5% of cases. There were 2.5% of late complications and 16% of early complications. Multivariate analysis identified six predictors for early complications: fistulotomy precut (OR=3.4, p=0.010), difficult cannulation (OR=21.5, p=0.002), attending’s procedural time (OR=2.4, p=0.020), choledocholithiasis (adjusted OR=1.8, p=0.015), cannulation time (adjusted OR=3.2, p=0.018) and ERCP duration (adjusted OR=2.7, p=0.041).

**Conclusion:**

Six risk factors for post-ERCP complications were identified. ERCP duration and cannulation time are suggested as new potential quality indicators.

## **RESUMO**

**Racional:**

A CPRE está associada a complicações, que podem ser prevenidas pelo reconhecimento de fatores de risco.

**Objetivo:**

Identificar tais fatores em nosso meio, com avaliação de qualidade.

**Métodos:**

Estudo retrospectivo em 194 pacientes de um hospital brasileiro, excluindo-se anatomia cirurgicamente alterada.

**Resultados:**

Executaram-se 211 CPREs: 97.6% foram terapêuticas, 83.4% iniciadas pelos residentes, com 89.6% de taxa de canulação profunda. Pré-corte foi necessário em 16.6% das CPREs e papilotomia clássica em 67.3%, com esvaziamento ductal de 74.5% em única sessão e 8.0% de falha técnica. Papilas inacessíveis ocorreram em 2.5% dos casos. Houve complicações tardias (2.5%) e precoces (16%). Em análise multivariada, identificaram-se seis preditores para complicações precoces: fistulotomia (RC=3.4, p=0.010), canulação difícil (RC=21.5, p=0.002), tempo do preceptor no procedimento (RC=2.4, p=0.020), coledocolitíase (RC ajustada=1.8, p=0.015), tempo de canulação (RC ajustada=3.2, p=0.018) e duração da CPRE (RC ajustada=2.7, p=0.041).

**Conclusão:**

Identificaram-se seis fatores para complicações pós-CPRE, sugerindo-se duração da CPRE e tempo de canulação como novos potenciais indicadores de qualidade.

## INTRODUCTION

Endoscopic retrograde cholangiopancreatography (ERCP) has evolved from a diagnostic test to an advanced therapeutic procedure requiring specific medical training^10^, which includes innovations such as the application of 3D simulators and ex-vivo models^**4**^. ERCP can treat biliary, pancreatic and ampullary diseases. However, there is a risk of unplanned events such as technical failure, complications and even hazards consequential to postural lesions, radiation or infection exposures^10^ ^,^ ^32^.

The procedure usually begins with wire-guided deep cannulation of the biliary tract or through precut. Suprapapillary needle-knife fistulotomy precut is characterized by an incision a few millimiters cephalad to the ostium, which is believed to reduce the risk of post-ERCP pancreatitis (PEP)^39^.

Post-ERCP complications can occur in the organs traversed by the endoscope, in distant organs such as the lungs, heart and kidneys or be subsequent to sedation in 5-40% of cases. PEP follows up to 15% of ERCPs, cholangitis 1-5%, duodenal perforationand hemorrhage up to 2%^3^ ^,^ ^10^ ^,^ ^32^ ^,^ ^17^ ^,^ ^35^.

Studies have identified that predisposing conditions for overall complications are coagulopathy/use of anticoagulants, acute cholangitis, surgical modification of the anatomy (Billroth II, Roux-en-Y), sphincterotomy, precut and previous failure to drain the biliary tract; and for sedation-related adverse events are cardiorespiratory comorbidities, advanced age, prolonged duration and prone position during the procedure^2^ ^,^ ^35^.

A landmark paper by Freeman et al. described several risk factors for PEP. The factors can be patient or procedure-related and behave sinergistically: female gender, young age, sphincter of Oddi dysfunction, previous pancreatitis, normal serum bilirubin, precut, pancreatic sphincterotomy, contrast injection in the pancreatic duct (PD), failure to insert a pancreatic stent and papillary balloon dilation. Difficult cannulation is also a risk factor for PEP and is defined as failure to deeply cannulate the biliary tract, after a predetermined interval and number of attempts (>3-5 guidewire passages or contrast injections in the PD)^8^ ^,^ ^16^ ^,^ ^27^ ^,^ ^36^ ^,^ ^38^ ^,^ ^40^.

The recognition of the risk factors associated with post-ERCP complications is very important as it may be able to allow risk stratification of the patients, to determine the need for hospital (re)admission and especially to implement preventive measures^17^ ^,^ ^22^. 

This study aims to identify risk factors for ERCP complications in a tertiary Brazilian center, with evaluation of quality indicators.

## METHODS

### Patients

This is an observational retrospective study, from November 2012 to November 2013, at a teaching tertiary care hospital part of the Brazilian Public Health System, Fortaleza, CE, Brazil. The institutional Ethics Committee approved the research protocol (registration 167.527) and all the participants signed informed consents. Participants included patients aged at least 16 years-old without surgically altered anatomy (Bilroth II or Roux-en-Y) undergoing ERCP at our facility.

All procedures were anesthesiologist-assisted with propofol-based sedation and continuous multiparameter monitoring. Therapeutic duodenoscopes and standard accessories were utilized. Prophylactic antibiotics (ciprofloxacin) were infused at the endoscopist’s discretion in patients with cholangitis, incomplete stone retrieval or failure to drain the biliary system.

### ERCP

Indications for ERCP were defined by preoperative evaluation with liver enzymes and ancillary examinations such as abdominal ultrasonography, computed tomography, magnetic resonance and, in some cases, endoscopic ultrasound.

Starting time was determinedat the moment the duodenoscope crossed the cricopharyngeus, with recording of total length and each physician’s (attending and/or trainee) proportional intervals in the procedure. Cannulation time was measured after the papilla was first touched by the instrument.

The endoscopists applied a minimally traumatic cannulation protocol in all subjects with native papilla, utilizing triple lumen sphincterotomes and 400cm-0.035inch hydrophilic guidewires. Pancreatic stents or rectal indomethacin were not available at the hospital and were not used in this study as PEP prophylaxis. Cannulation was characterized by free and deep instrumentation of the common bile duct (CBD). Cholangiography without deep instrumentation was deemed unsuccessful.

Trainees started most of the ERCPs, were allotted 10 min for cannulation and, if they failed, the attending would take over. Futhermore, at the attending’s discretion, aliquots of intrapapillary contrast injection were used. Fistulotomy precut was performed following the attending’s failure in cannulation by standard means (i.e. difficult cannulation) in therapeutic ERCPs with intact papillas and common bile duct diameter of at least 10 mm.

According to established criteria^3^ ^,^ ^10^ ^,^ ^35^, this investigation used the following definitions:

#### *Technical failure*

Inability to progress the guidewire through a stenosis, complete the succeeding maneuvers or cannulate the duct, excluding inacessiblepapillas. In case of technical failure, the attending physician would re-attempt the ERCP in a second time.

#### *Complications*

ERCPattributable adverse effects requiring hospital admission or prolongation of actual admission, identified by in-person and phone follow-up in the first, 7^th^ and 30^th^ post-procedural days, classified as: 1) early: onset in less than 24 h; 2) late: onset in 8-30 days; 3) mild: up to three days of hospital stay; 4) severe: >10 days of hospital stay, need of invasive therapy, surgery, admission in intensive care unit or fatality; 5) PEP: typical abdominal pain associated with amylase or lipase elevation of at least three times the normal value measured 24 h after the ERCP; 6) bleeding: clinical (not endoscopic), categorized as mild if transfusion was not needed and severe otherwise; 7) duodenal perforation: tomographic diagnosis, with the presence of luminal liquid, contrast extravasation, intraperitoneal or retroperitoneal air.

### Statistical analysis

SPSS 20 (IBM Corporation, Armonk, NY, USA) was utilized to process the data; Student’s t-test for the numeric variables (time-related); calculation of standard deviations, averages and medians, with the latter established as statistical cut-offs, since they represent the 50^th^ percentile. In the bivariate analysis of the risk factors for early and late complications and for PEP, the methods of chi-square and likelihood ratio were applied, with calculation of OR and their CI95%. P< 0.05 was deemed significant.The studied variables comprised gender, age, indication, sphincterotomy, precut, stone retrieval, endoscopist, prophylactic antibiotics, technical failure, duration, cannulation time, number of guidewire passages and contrast injections in the PD. Considering complications as a dichotomic variable, a stepwise multiple logistic regression with backward approach was executed with the variables of p<0.2 at bivariate level, in order to determine the independent risk factors, with respective adjusted OR and CI95%.

## RESULTS

A total of 211 ERCPs were performed in 194 patients, with mean age of 54±18.9 years (16-91 years), 79.1% were female and 20.9% male. Most of the ERCPs (97.6%) were therapeutic, with the main indications of choledocholithiasis (57.8%) and suspected choledocholithiasis (16.6%), including acute cholangitis (5.7%) and on-going acute pancreatitis (1.6%).

### Quality indicators

Table 1 depicts the results of the performed ERCPs. Trainees started the procedures in 83.4% of the cases. The achieved deep cannulation rate was 89.6%, with precut needed in 16.6% and classic sphincterotomyin 67.3%. Undesired contrast injection throughout the PD occurred in 12.8% and guidewire passage occurred in 34.1% of all ERCPs. Inacessiblepapillas were present in 2.5% of cases due to duodenal (1.5%) and pyloric strictures (0.5%) or intradiverticular location (0.5%).

**TABLE 1:** Results of all ERCPs performed

	n	(%)	Mean ± SD
Deepcannulation	189	(89.6)	
Sphincterotomy	142	(67.3)	
Precut	35	(16.6)	
Endoscopist Trainee Attending Both	50 35 126	(23.7) (16.6) (59.7)	
Number of undesired guidewire passage in PD 0 1 to 5 6 to 20	139 63 9	(65.9) (29.9) (4.2)	1.1±2.5
Number of undesired contrast injections in PD 0 1 2 to 5	184 22 5	(87.2) (10.4) (2.4)	0.17±0.5
Technical failure In cannulation In guidewire’s progression	17 13 4	(8.0) (6.1) (1.9)	
Prophylacticantibiotics	12	(5.7)	
Time (min) ERCP duration Trainee Attending Cannulation			38.3±20.0 18.2±13.7 19.9±20.8 10.3±10.0

PD=pancreatic duct, SD=standard deviation

Table 2 presents the profiles of ERCPs due to choledocholithiasis. Most of the biliary stones were sized more than 10 mm (55.7%), located in the common bile duct (95.9%), and completely extracted in 75.4% of the cases with standard accessories (baskets, balloons and mechanical lithotriptor). 

**TABLE 2:** Profile of ERCPs due to choledocholithiasis

	n	(%)
Stone size (mm) <10 >10	54 68	(44.3) (55.7)
Stone location CBD CBD and intrahepatic ducts Others	117 3 2	(95.9) (2.4) (1.7)
Extraction devices Balloon Basket Balloon and basket Lithotripsy	60 17 42 3	(49.1) (13.9) (34.5) (2.5)
Stone Extraction Complete Incomplete	92 30	(75.4) (24.6)

CBD=common bile duct

The early (16.0%) and late (2.5%) post-ERCP complications are shown in Table 3. PEP was most frequent (6.5%), followed by infection (3%), comprising cholangitis (2.0%), acute cholecystitis (0.5%) and bacteremia (0.5%). Sedation-related adverse events (2.0%) were characterized by intraprocedural oxygen desaturation (1.5%) and headache (0.5%). The late complications included cholecystitis (1.0%), pneumonia (1.0%) and abdominal abscess (0.5%).

**TABLE 3:** Early and late-onset post-ERCP complications

	Earlycomplications n(%)			Late complications n(%)	
Acutepancreatitis Mild Severe	13 11 2	(6.5) (5.5) (1.0)	Cholecystitis	2	(1.0)
Infection	6	(3.0)	Pneumonia	2	(1.0)
Sedation	4	(2.0)	Abdominal abscess	1	(0.5)
Bleeding Mild Severe	3 1 2	(1.5) (0.5) (1.0)			
Fatality	3	(1.5)			
Basket’simpaction	1	(0.5)			
Paralyticileus	1	(0.5)			
Duodenal perforation	1	(0.5)			
Total	32	(16.0)		5	(2.5)

Some serious complications were observed: fatality (1.5%), duodenal perforation (0.5%) and Dormia basket impaction (0.5%). The fatalities resulted from necrotizing PEP (1%) and refractory post-sphincterotomy bleeding (0.5%) in patients with many severe comorbities. The other two complications were considered serious given the need of surgical intervention. The latter occurred during attempted removal of a large hard stone, with fracture of the basket’s metallic wire (it was severed by the mechanical lithotripter).

### Risk factors for post-ERCP complications

As demonstrated in Table 4, only risk factors for early complications were isolated given that none of the potential predictors for late complications achieved statistical significance. The bivariate analysis obtained six risk factors: the three first for PEP and the other for overall complications. The cut-off numbers (in minutes) represent the calculated medians for the numeric variables. The independent risk factors for early complications resulted from the multiple logistic regression model taken from the pool of determinants that reached a p value cut-off <0.2 in the bivariate analysis (nine potential factors for early and three for late complications). In this study gender, age, sphincterotomy, stone extraction (complete or incomplete), the use of prophylactic antibiotics and technical failure did not prove to be statistically related to the development of complications.

**TABLE 4:** Dependent and independent risk factors for early post-ERCP complications

Dependent risk factors				Independent risk factors			
	OR	CI95%	p		Adjusted OR*	CI95%	p
Fistulotomyprecut	3.4	1.1 - 10.4	0.010	Choledocholithiasis	1.8	1.1 - 3.0	0.015
>5 undesired guidewire passages in PD	5.0	0.8 - 28.7	0.047	ERCP duration>34 min (mean)	2.7	1.1 - 6.8	0.041
>1 contrastinjections in PD	21.5	3.2 -142.7	0.002	Cannulation time >7 min (mean)	3.2	1.2 - 8.2	0.018
ERCP duration>34min (mean)	2.6	1.2 - 5.7	0.012				
Attending’s procedural time >15min (mean)	2.4	1.1 - 5.2	0.020				
Cannulation time >7min (mean)	3.4	1.5 - 7.8	0.002				

PD=pancreatic duct; *adjusted to gender, indication, precut, duration, attending’s procedural time, cannulation time

## DISCUSSION

Despite the variable design of other studies, post-ERCP complications have typically ranged between 4-16%, with most of the data focusing on early complications. However, an assessment of patients in the 30^th^ post-procedural day can aid in documentation and estimation of the late complications, which are relatively understudied^11^ ^,^ ^21^ ^,^ ^22^ ^,^ ^26^ ^,^ ^29^. Unlike the majority of previous research, we report both early (16%) and late (2.5%) complication rates, using these results to identify risk factors for post-ERCP complications in our center.

Globally over the last decade, ERCP quality indicators have been analyzed more closely and professional entities such as American Society for Gastrointestinal Endoscopy (ASGE), American College of Gastroenterology (ACG) and World Endoscopy Organization (WEO) have released helpful practice guidelines or statements on the matter^1^ ^,^ ^15^. However, in light of the great variance of intraprocedural quality internationally, as suggested by a recent meta-analysis^12^, we felt it is necessary to conduct an evaluation on quality metrics for our own center. 

In 2015, an ASGE-ACG joint taskforce revised the guideline initially proposed in 2006. The updated performance aims were: appropriate indication in over 90% of cases, deep cannulation >90% in intact papillas, removal of stones sized up to 10 mm>90% (the number of sessions not specified), perforation <0.2% and bleeding<1%, without any goals for PEP, precut and infectious complications. On the other hand, WEO’s statement acknowledges cannulation rates of over 90-95%, PEP of 1-7%, without goals for other complications, nor for precut. It is necessary to emphasize that all those performance marks refer to experienced endoscopists, not trainees^1^ ^,^ ^12^ ^,^ ^15^.

Ideally, the suggested ERCP quality metrics should be separated by complexity. Currently, the most accepted scale is the modified Schutz andAbbott´s, which classified maneuvers as extraction <10mm stones as easy and ones sized>10 mm as intermediate^12^ ^,^ ^13^ ^,^ ^28^.

In this study, 97.6% of the ERCPs were therapeutic and only 2.4% diagnostic, with standard indications, especially choledocholithiasis (74.4%, suspected and confirmed), as endorsed by ASGE^1^. Most (55.7%) of the removed stones were large (>10 mm), i. e. classified as Schutz and Abbott’s intermediate difficult, with 74.5% of complete extraction at single session. Given the lack of proposed goals by the taskforce referring to large stones, we can only compare to other Brazilian cohorts, which presented alike ratios^11^ ^,^ ^22^.

Our deep cannulation index was 89.6%, with sphincterotomy in 67.3% and precut in 16.6% of cases. Most of the procedures occurred without guidewire passage (65.9%) or opacification of the PD(87.2%). A Chilean study reported 76% cannulation, 51% sphincterotomy, 16% precut and 26% PD opacification, attaining inferior results^21^. Correlating with the quality guidelines, our cannulation index is slightly different, which, in an academic setting, can partially be explained by the learning curve of the trainees^14^.

The current patient cohort presented 8% of technical failure, congruent with previous data, which generally report failure in the 5-20% range^30^ ^,^ ^32^ ^,^ ^34^. Although not a quality indicator per se, this parameter can be used to indirectly aid in assessing the endoscopist’s performance. Amongst the many treatment plans following unsuccessful ERCPs, a very efficient strategy is to re-attempt it, either by the same professionals (as adopted by our center) or transfer to another referral hospital^30^ ^,^ ^34^.

In this study, the main early complications described were PEP (6.5%), infection (3.0%) and bleeding (1.5%). In addition to the infrequent but severe ones as basket impaction (0.5%), duodenal perforation (0.5%), and mortality (1.5%).

The most frequent adverse effect arising from ERCP is PEP, followed by cholangitis and bleeding^1^ ^-^ ^3^ ^,^ ^32^ ^,^ ^35^. Our complication statistics have met the quality metrics only for PEP. Nevertheless, when matched with other Brazilian and South American data, all of them are within expected percentages: PEP (1.5-11.5%), bleeding (1-3.1%), cholangitis (1.1-4.2%), perforation (0.6-2.1%) and mortality (0-2.1%)^11^ ^,^ ^21^ ^,^ ^22^ ^,^ ^26^ ^,^ ^29^.

Post-ERCP perforation can occur in the duodenal wall (endoscope related), in the periampullary area (sphincterotomy related), in the ducts or be characterized by the presence of retroperitoneal air. Treatment depends on the location, clinical status and radiographic imaging^2^. In this investigation, the perforation was duodenal, with surgical management.

A rather infrequent complication, Dormia basket impaction (with captured stone and fracture of the traction wire), is usually described in 0.8-6% of ERCPs, with large and hard stones, in patients with disproportional distal common bile duct diameter. Surgery is considered the last resort after failure of endoscopic attempts. Recently, sphincteroplasty has shown good results in such cases, avoiding these complications^19^ ^,^ ^33^.

Worldwide, ERCP mortality ranges from 0-1.5% and can result from any complication. It is generally higher in therapeutic procedures^2^ ^,^ ^3^ ^,^ ^35^. In this series, it was secondary to severe PEP in two patients and to post-sphincterotomy hemorrhage in one, in therapeutic ERCPs. All the patients had serious comorbidities. 

Furthermore, this investigation also presented late complications, namely cholecystitis (1%), abdominal abscess (0.5%) and pneumonia (1%). Even though the authors did not explore such correlation, the latter is likely related to hypoxemia or bronchial aspiration secondary to oversedation and prone position of the patient. Hence, consideration should be given for tracheal intubation during procedures in high risk patients.

The data regarding late post-ERCP undesired events is relatively scant. In Brazil, at least to the best of the authors’ knowledge, there is only one other (than the present) article that explored them, through phone follow-up, but failing to clearly classify them^22^. International literature cites mainly infection, which can be as high as 6-24%, in addition to longer-term complications such as papillary stenosis and stones recurrence^3^ ^,^ ^7^ ^,^ ^35^.

As the indications for ERCP have risen, a greater focus on recognizing and preventing undesired events has emerged. Several trials have evaluated risk factors for complications, but their relative contribution to post-ERCP morbidity and mortality is unknown. Nevertheless, their identification can be used to distinguish patients at highest risk, for whom ERCP should be avoided if possible or in which protective endoscopic and pharmacologic interventions might be considered^8^ ^,^ ^17^.

Although relevant studies are heterogeneous and sometimes omit potential key risk factors, important patterns are apparent^18^. The literature general consensus on risk factors for PEP include young age, female gender, normal serum bilirubin, previous pancreatitis, sphincter of Oddi dysfunction, difficult cannulation, PD opacification, precut, manometry and pancreatic/minor papilla sphincterotomy^24^ ^,^ ^41^. 

The risk factors are believed to exert a cumulative effect. In fact, Jeurnink et al attributed a sum score to each risk factor present in a given patient, creating a prognostic model and categorizing patients into high-risk (>3 factors) or low-risk (up to 3 factors) for complications, with recommendation for overnight observation in the first group^17^.

Our bivariate analysis resulted in the following technique-related risk factors: >5 undesired passages of the guidewire in the PD (OR=5.0, p=0.047), >1 contrast injection in the PD (OR=21.5, p=0.002); i.e., difficult cannulation, and fistulotomy precut (OR=3.4, p=0.010). We also demonstrated that intrapancreatic contrast offers quadrupled odds to PEP than guidewire, in agreement with previous articles and meta-analysis^2^ ^,^ ^5^ ^,^ ^8^ ^,^ ^9^ ^,^ ^16^ ^,^ ^27^ ^,^ ^37^ ^,^ ^40^. In practice, despite not formally evaluated yet, many endoscopists (including ours) adopted an hybrid technique combining guidewire and minimal contrast use to delineate the ductal course^18^ ^,^ ^41^. 

Precut, regardless of being widely utilized in difficult cannulation, has an unclear relationship to complications. Some articles classify it as independent risk factor for PEP, while others demonstrate the same figures of conventional sphincterotomy. The only existing consensus regards the timing of execution, after failure of ordinary cannulation methods^5^ ^,^ ^6^ ^,^ ^31^ ^,^ ^38^ ^-^ ^40^.

However, all those data usually refer to standard technique precut, characterized by an incision starting a few millimeters above the papillary ostium and progressing cephalad to expose the common bile duct opening. In fistulotomy precut, the dissection begins above the ostium and extends downwards. Recently, pancreatic precut has been described. Nevertheless, there are no reports comparing the outcomes of each variant^6^ ^,^ ^31^ ^,^ ^39^. In this study, fistulotomy precut, our only executed variant, was a dependent predictor for PEP. 

The only indication previously associated with post-ERCP complications is sphincter of Oddi dysfunction^2^ ^,^ ^3^ ^,^ ^8^ ^,^ ^16^ ^,^ ^17^ ^,^ ^27^ ^,^ ^35^ ^,^ ^40^. We described choledocholithiasis as another independent risk factor, with almost doubled odds for complications (adjusted OR=1.8, p=0.015). However, the authors acknowledge the possibility of a selection bias, as it was the most frequent diagnosis in this investigation.

The other independent predictors proposed in this case series are mean ERCP’s length >34 min (adjusted OR=2.7, p=0.041) and mean cannulation time >7 min (adjusted OR=3.2. p=0.018), both implicating in almost three times the chance of adverse effects.

Articles analyzing time as a technical factor are scant. In one of the few published, Metha et al., conflicting with us, demonstrated that the procedure´s length does not influence the complications, except for post-ERCP bleeding^23^.

Up until now, there is not any data linking cannulation time with post-ERCP complications. Previously, it was assessed and recommended by Tian et al. as a more objective manner to interpret technical difficulty (rather than cannulation attempts)^36^.

The present authors would like to propose cannulation time up to 7 min and ERCP duration up to 34 min as new potential quality parameters. Theoretically, longer procedures can either be of higher complexity or unsuccessful. Hence, the suggested timespans afford reasonable time to accomplish the intended intervention or to change the approach. We believe that their most useful practical application is in training settings, with expected reduction of complications. In fact, in a recent randomized controlled trial, Pan etal. suggested a 10 min interval for trainees to attempt biliary cannulation^25^.

 The limitations of this study include a single center’s sample, with possibility of a selection bias caused by the hospital being a referral unit. Its main strength was the establishment of ERCP duration and cannulation time as independent risks factors for post-ERCP complications, along with the description of late undesired events, such as pneumonia. We also provide rationale for further assessment regarding time measurement and its influence on ERCP’s outcomes.

Another highlight was demonstration of fistulotomy precut as a dependent predictor for PEP, but at approximately seven times less odds than PD opacification. This series is also critical within our country as the first assessment of ERCP quality performance that has led to two new potential quality metrics.

## CONCLUSION

Risk factors for post-ERCP complications are difficult cannulation, fistulotomy precut, attending’s procedural time longer than 15 min, choledocholithiasis, cannulation time longer than 7 min and ERCP’s duration longer than 34 min.ERCP duration and cannulation time are newly described risk factors for post-ERCP complications and we would like to recommend them as new potential quality indicators, with respective cut-offs.
